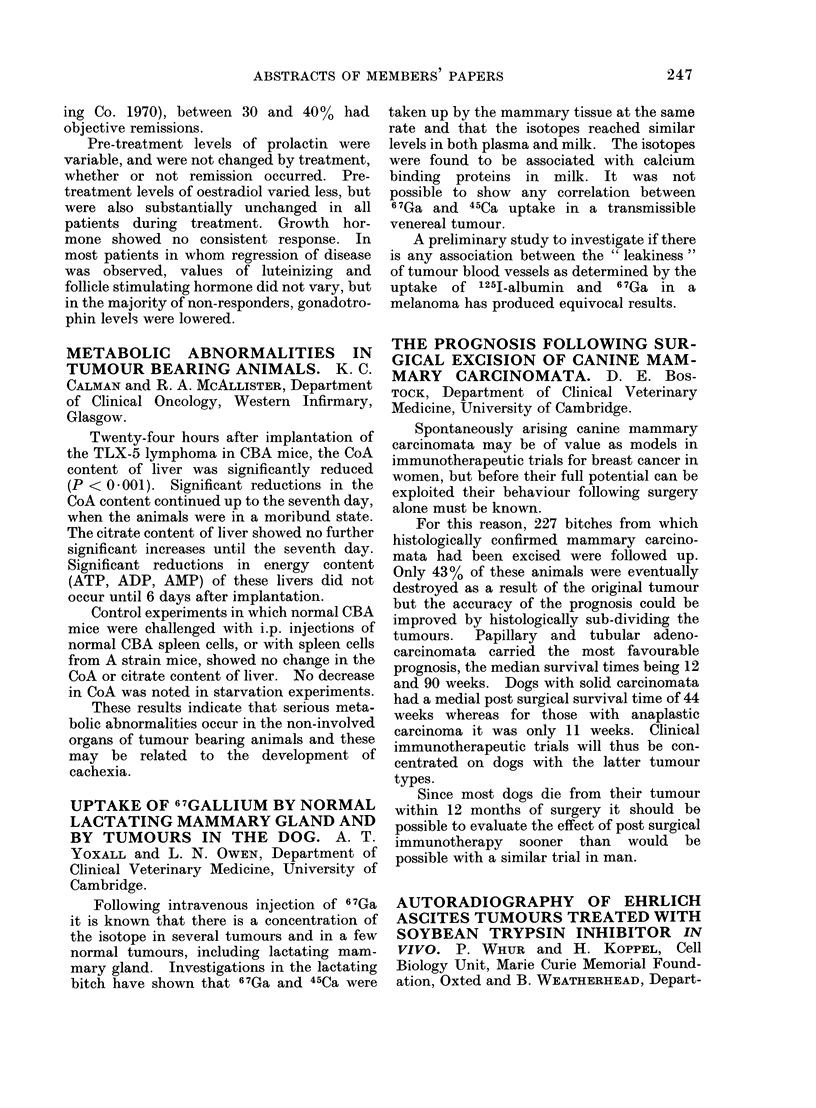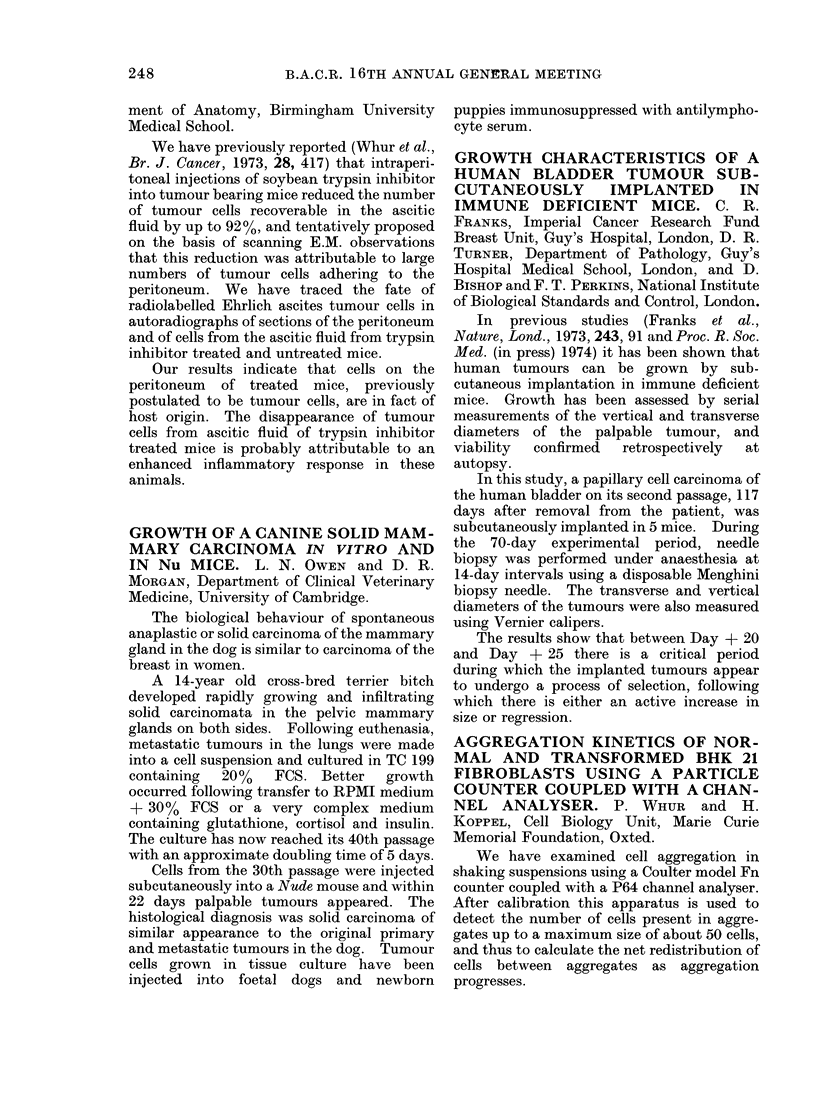# Proceedings: Autoradiography of Ehrlich ascites tumours treated with soybean trypsin inhibitor in vivo.

**DOI:** 10.1038/bjc.1975.180

**Published:** 1975-08

**Authors:** P. Whur, H. Koppel, B. Weatherhead


**AUTORADIOGRAPHY OF EHRLICH ASCITES TUMOURS TREATED WITH SOYBEAN TRYPSIN INHIBITOR *IN VIVO*.** P. Whur and H. Koppel, Cell Biology Unit, Marie Curie Memorial Foundation, Oxted and B. Weatherhead, Department of Anatomy, Birmingham University Medical School.

We have previously reported (Whur *et al.*, *Br. J. Cancer*, 1973, **28**, 417) that intraperitoneal injections of soybean trypsin inhibitor into tumour bearing mice reduced the number of tumour cells recoverable in the ascitic fluid by up to 92%, and tentatively proposed on the basis of scanning E.M. observations that this reduction was attributable to large numbers of tumour cells adhering to the peritoneum. We have traced the fate of radiolabelled Ehrlich ascites tumour cells in autoradiographs of sections of the peritoneum and of cells from the ascitic fluid from trypsin inhibitor treated and untreated mice.

Our results indicate that cells on the peritoneum of treated mice, previously postulated to be tumour cells, are in fact of host origin. The disappearance of tumour cells from ascitic fluid of trypsin inhibitor treated mice is probably attributable to an enhanced inflammatory response in these animals.